# A TIR-NBS-LRR Gene *MdTNL1* Regulates Resistance to Glomerella Leaf Spot in Apple

**DOI:** 10.3390/ijms23116323

**Published:** 2022-06-05

**Authors:** Lingling Lv, Yingshuang Liu, Suhua Bai, Khurshid Sadullaevich Turakulov, Chaohua Dong, Yugang Zhang

**Affiliations:** 1 Engineering Laboratory of Genetic Improvement of Horticultural Crops of Shandong Province, Qingdao Agricultural University, Qingdao 266109, China; 201301014@qau.edu.cn (L.L.); dongchaohua@qau.edu.cn (C.D.); 2 College of Horticulture, Qingdao Agricultural University, Qingdao 266109, China; liuyingshuang@stu.qau.edu.cn; 3 College of Life Sciences, Qingdao Agricultural University, Qingdao 266109, China; shbai@qau.edu.cn; 4 Institute of Genetics and Plant Experimental Biology, Academy Sciences of Uzbekistan, Tashkent 111226, Uzbekistan; sadullaevich@yahoo.com

**Keywords:** *Malus domestica*, *C. fructicola*, NBS-LRR gene, fungal disease resistance, plant immunity

## Abstract

Glomerella leaf spot (GLS), caused by the fungus *Colletotrichum fructicola*, is one of the most devastating apple diseases. Our previous study reported that the GLS resistance locus was defined on the chromosome 15 region. Here, we further found a single-nucleotide polymorphism (SNP) site (SNP_7309212_) in the GLS resistance that was able to distinguish resistant cultivars (lines) from susceptible ones. On the basis of the SNP site, we cloned a TNL gene from the GLS resistant locus and named it *MdTNL1* (NCBI Accession Number: ON402514). This gene contains a toll/interleukin-1 receptor transmembrane domain (TIR), nucleotide-binding sites (NBS), and leucine-rich repeat (LRR) domain. Subcellular location indicated that MdTNL1 was expressed in the nucleus and cell membrane. Ectopic overexpression of *MdTNL1* in *Nicotiana benthamiana* caused cell death. We further demonstrated allelic polymorphisms in *MdTNL1*. It is noteworthy that NBS and LRR domains of the MdTNL1 protein serve as the repository for generating allelic diversity. Quantitative real-time PCR (qRT-PCR) assay revealed that *MdTNL1* was highly expressed in resistant apple cultivar ‘Fuji’ after inoculation with *C. fructicola,* whereas susceptible cultivar ‘Golden Delicious’ exhibited low expression after inoculation. Over-expression of *MdTNL1-1* in susceptible apple fruits and leaves improved disease resistance, while in ‘Orin’ calli, silencing the *MdTNL1-1* gene conversely decreased GLS resistance. In conclusion, we identified a GLS associated with SNP_7309212_ and demonstrated that a TIR-NBS-LRR gene *MdTNL1-1* positively regulates GLS resistance in apple.

## 1. Introduction

Glomerella leaf spot (GLS) is a severe apple disease caused by *Colletotrichum fructicola*, which has affected the industrial development of apple (*Malus domestica*) in the last decades [1,2]. GLS mainly occurred in some major apple cultivars, such as ‘Gala’ and ‘Golden Delicious’. Some other widely cultured apple cultivars, such as ‘Fuji’ and its derivative cultivars, exhibited high resistance to GLS [3]. GLS was first reported in the United States in 1970, but it did not come to attention for the less damage to the apple production [4]. In China, GLS was first found in Henan province, in the middle area of China, in 2008 [2]. Climate conditions of high temperature and high humidity in this area are suitable for GLS occurrence. Once the disease occurs, it develops rapidly, leading to necrosis spots in the leaves followed by defoliation of leaves [5]. In 2013, the ‘Gala’ orchard in Xianyang City, China, suffered from leaf necrosis with an area of 1309 hm^2^, which is a typical manifestation of GLS. In 2018, more than 70% of GLS diseases occurred in Wugong County, China. GLS also weakens tree vigor, causes fruit necrotic lesions, decreases fruit quality, and affects the fruit growth and the fruit setting rate of the next year [6,7]. For its rapid development and the difficulty of prevention, GLS has become an epidemic fungal disease that seriously threatens the apple industry in China [8]. For effective management of this fungal disease, it is necessary to explore the defense mechanism of resistant apple cultivars. Previous studies explored the apple defense mechanism against GLS. MicroRNA *Md-miRln20* negatively regulates the resistance to GLS by suppressing *Md-TN1-GLS* expression [9]. MKK4-MPK3-WRKY17-mediated salicylic acid degradation is also the regulation of the GLS resistance [10]. Although these studies revealed the regulation of apple resistance to GLS, the resistance gene specific to GLS remains to be identified.

Nucleotide binding sites and leucine-rich repeats genes (NBS-LRRs or NLRs) constitute a large family that plays a crucial role in plant resistance to bacterial and fungal pathogens [11]. By interacting with effectors of pathogens, NLRs trigger effector-triggered immunity (ETI), leading pathogen-specific immune responses [12,13]. NLR proteins can be divided into two classes on the basis of their N-terminal domains, including the toll/interleukin-1 receptor (TIR) for NLRs proteins (TNL) and the coiled-coil (CC) for NLRs proteins (CNL) [14]. TNL have been identified as resistant genes in *Arabidopsis thaliana* [15], grape (*Vitis vinifera*) [16], rice (*Oryza sativa*) [17], and cotton (*Gossypium hirsutum*). In apple plants, the resistance locus Rvi15 (Vr2) for apple scab has been found to contain three TNL genes [18]. The quantitative trait locus for resistance to fire blight in apple is linked to NLR gene [19], and *powdery mildew* resistance gene *Pl1* is also linked to TNS genes [20]. However, whether the TNL gene is the key factor regulating apple GLS resistance remains to be known.

Our previous research has reported that apple GLS resistance is controlled by a recessive single gene on chromosome 15 [3], and the resistance gene loci (Rgls) are located between SNP_4208_ and SNP_4257_, as determined by high-resolution melting (HRM) technique [21]. However, the major recessive gene has not been cloned. In the present study, we cloned the gene responsible for GLS resistance on the basis of the SNP analysis and further addressed its function in GLS resistance.

## 2. Results

### 2.1. GLS Resistance Evaluation of Apple Cultivars

**Figure 1:**
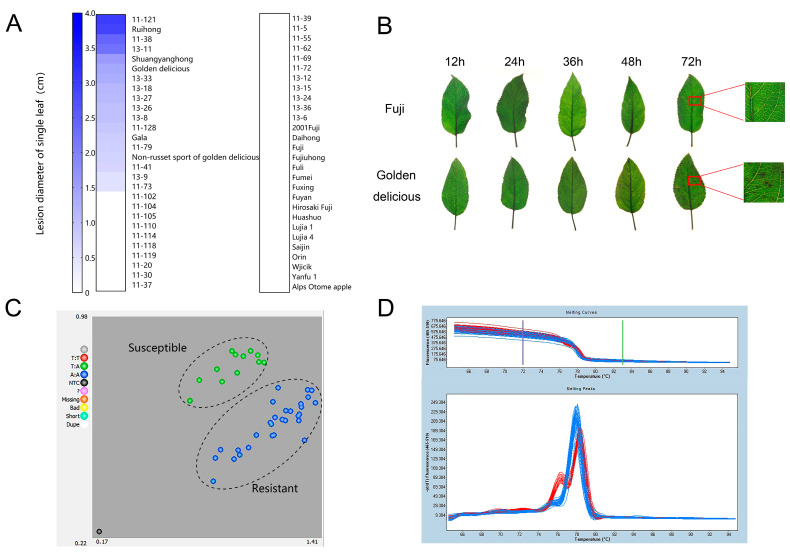
Resistance identification of different apple cultivars to GLS and genotyping of apple cultivars (lines) with different resistance levels using SNP_7309212_. (**A**) Lesion diameter on the single leaf (cm) of apple. A total of 56 cultivars (lines) were evaluated, including 34 F1 hybrid populations from ‘Fuji’ × ‘Golden delicious’ and 22 cultivars. (**B**) Symptoms of disease-resistant cultivar ‘Fuji’ and susceptible cultivar ‘Golden Delicious’ after inoculated with *C. fructicola.* (**C**) Distribution of SNP_7309212_ genotype by KASP analysis. Red solid circles indicate homozygous TT, blue ones represent homozygous AA, and green are heterozygous AT. (**D**) SNP_7309212_ genotype assay of apple cultivars (lines) with different disease resistance using HRM technique marked. The blue melting curves indicate resistant apples cultivars (lines), and red melting curves represent susceptible cultivars (lines).

For KASP analysis, we evaluated the GLS resistance of 56 apple germplasms, including 22 common cultivars and 34 F1 hybrids from a cross of ‘Fuji’ with ‘Golden Delicious’. The spore of *C. fructicola* were dotted on the detached leaves of the 56 apple germplasms for inoculation. The lesion diameters of the inoculated leaves were measured 3 days after inoculation. Five cultivars that exhibited obvious lesions including ‘Golden Delicious’, ‘Non-russet sport of Golden Delicious’, ‘Ruihong’, ‘Shuangyanghong’, and ‘Gala’ were susceptible to GLS. The other 17 cultivars showed high resistance to *C. fructicola* (Figure 1A and Figure S1; Table S1). Among the 34 F1 hybrids, 14 lines exhibited susceptible disease and 21 lines were resistant to GLS (Figure 1A and Figure S1; Table S1). By spraying conidia suspension of C. *fructicola* on the leaves of susceptible cultivar ‘Golden Delicious’, visible lesions appeared on the leaves of ‘Golden Delicious’ 36 h after inoculation. There are about 40 necrosis spots per cm^2^ of leaf area 72 h after inoculation. No visible necrotic lesions were observed on the leaves of ‘Fuji’ 72 h after inoculation, indicating that ‘Fuji’ is highly resistant to GLS (Figure 1B and Figure S2A).

### 2.2. SNP_7309212_ Sites Show Polymorphism in Resistant and Susceptible Apple Germplasms

On the basis of the previous finding that the resistance gene was located at the 4.1–4.6 Mb candidate region of chromosome 15 by whole-genome resequencing [1], we looked for SNP sites in this region by KASP sequencing using the susceptible and resistant cultivars evaluated above. According to Liu’s results [1], we selected 17 SNP sites in the 4.1–4.6 Mb candidate region (Table S2). KASP detection on 17 SNP sites followed the random using of resistant and susceptible materials for 17 SNP sites (Tables S3 and S4). We found that among these SNP sites, only one SNP site was different between resistant and susceptible leaves. This SNP (designate as SNP_7309212_) was inferred to be closely associated with GLS resistance. Among the examined apple germplasms, there are two genotypes at SNP_7309212_, namely, homozygous genotype AA and heterozygous genotype TA, which correspond exactly to resistant and susceptible germplasms. It is noted that all AA genotypes were clustered together, while TT genotypes were clustered together with heterozygous genotype TA (Figure 1C; Table S4). PCR amplification and sequencing confirmed the resistant/sensitive pattern represented by A/T (isoleucine/phenylalanine) (Table S5). The SNP_7309212_ locus appeared different between disease-resistant and susceptible apple germplasms. In addition, high-resolution melting (HRM) analysis showed that obviously different melting curves were observed between resistant and susceptible germplasms at SNP_7309212_ (Figure 1D). These results indicated that SNP_7309212_ marker is closely associated with GLS resistance in apple.

### 2.3. Cloning and Characterization of GLS-Resistant Gene

**Figure 2:**
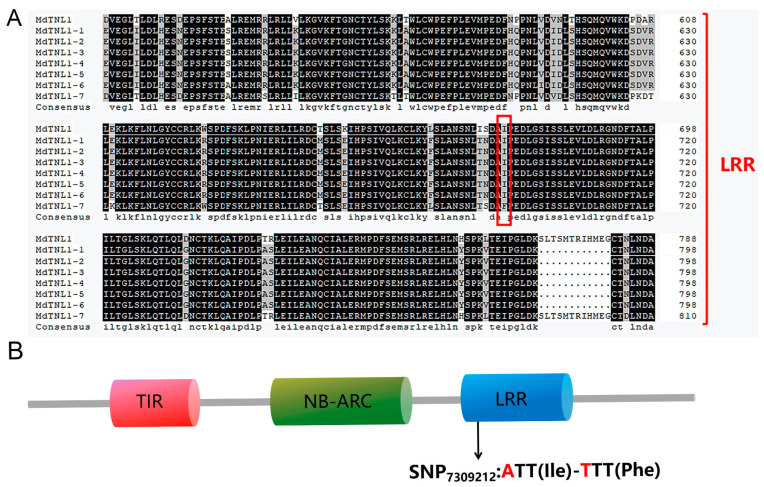
Amino acid sequence and domain constitution of MdTNL1. (**A**) Multiple sequence alignment of LRR domain of MdTNL1 proteins in apple. The red box represents the amino acid of **SNP_7309212_**. (**B**) Conserved domain of MdTNL1 predicted by SMART.

**Figure 3:**
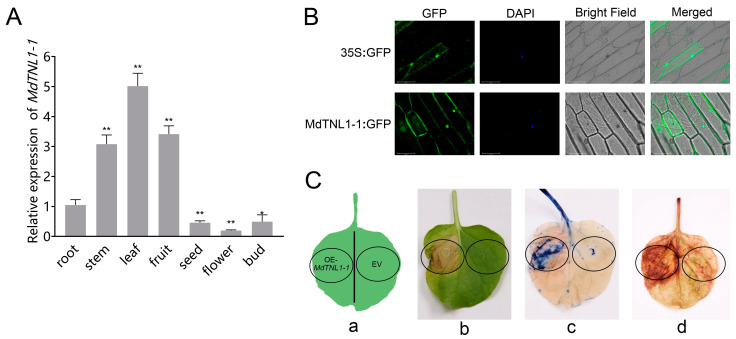
Expression analysis of *MdTNL1-1*. (**A**) Differential expression of *MdTNL1-1* in ‘Fuji’ apple tissues. Data are presented as the mean ± SD of three biological replicates. Asterisks indicate significant difference compared to root (* *p* < 0.05; ** *p* < 0.01, one-way ANOVA test by Tukey’s test). **(B**) Subcellular localization of MdTNL1-1 protein. Full length CDS was fused with green fluorescent protein (GFP) and expressed in onion epidermal cells. Fluorescence was examined using confocal laser microscopy. Bars, 100 μm. (**C**) Transient expression of *MdTNL1-1* in *Nicotiana benthamiana* (tobacco) leaves. EV, infiltrated with *Agrobacterium* containing empty vector, pCAMBIA1300. OE-*MdTNL1-1*, infiltrated with *Agrobacterium* harboring pCAMBIA1300-*MdTNL1-1*. (**a**) Injection site of leaf. (**b**) Phenotype after 7 days of leaf injection. (**c**) Typan blue staining (TPB) of tobacco leaves 30 min after infiltration. (**d**) Diaminobenzidine staining (DAB) of tobacco leaves 4 h after infiltration.

We cloned a *MdTNL1* gene from ‘Golden Delicious’ and ‘Fuji’ and obtained seven variants (*MdTNL1-1*–*MdTNL1-7*). Most nucleotides of the seven variants were identical, except several single nucleotide sites and a fragment of 3′ terminal, which showed sequence polymorphism between the seven variants (Figure 2A). A and T base substitutions occurred at the SNP_7309212_ sites of *MdTNL1-7*, resulting in nonsynonymous amino acid substitutions (I/F) at this site (Figure 2A). Among them, *MdTNL1-1* exists only in ‘Fuji’, and *MdTNL1-7* appears only in ‘Golden Delicious’, whereas *MdTNL1-2-MdTNL1-6* sequences were found in both cultivars. Domain analysis revealed that MdTNL1 protein, which was similar to the reported TNLs in different species, contains three major conserved domains: N-terminal TIR, NB-ARC, and C-terminal LRR (Figure 2B). *MdTNL1-1* sequence (NCBI Accession Number: ON402514) was chosen as the target sequence *MdTNL1* for further research. It encoded an 880 amino acid protein, and the full-length CDS consisted of 2640 bp. qRT-PCR analysis demonstrated that in apple, the expression level of *MdTNL1-1* was highest in leaves, followed by fruits, and the lowest in flowers (Figure 3A). Subcellular localization showed that the MdTNL1-1-GFP fusion protein was localized at the nucleus and plasma membrane (Figure 3B). In addition, *MdTNL1* expression was significantly upregulated in ‘Fuji’ after inoculation but was suppressed in ‘Golden Delicious’ (Figure S2B). These results suggest that the level of *MdTNL1* expression is associated with GLS resistance in apple. In addition, overexpression of *MdTNL1-1* in the leaves of *Nicotiana benthamiana* for 7 days caused tissue necrosis in tobacco leaves, which was confirmed by the trypan blue staining. DAB staining showed the H_2_O_2_ accumulation at the necrotic sites of tobacco (Figure 3C).

### 2.4. MdTNL1-1 Was Required for the Resistance of Apple Plant to C. fructicola

**Figure 4:**
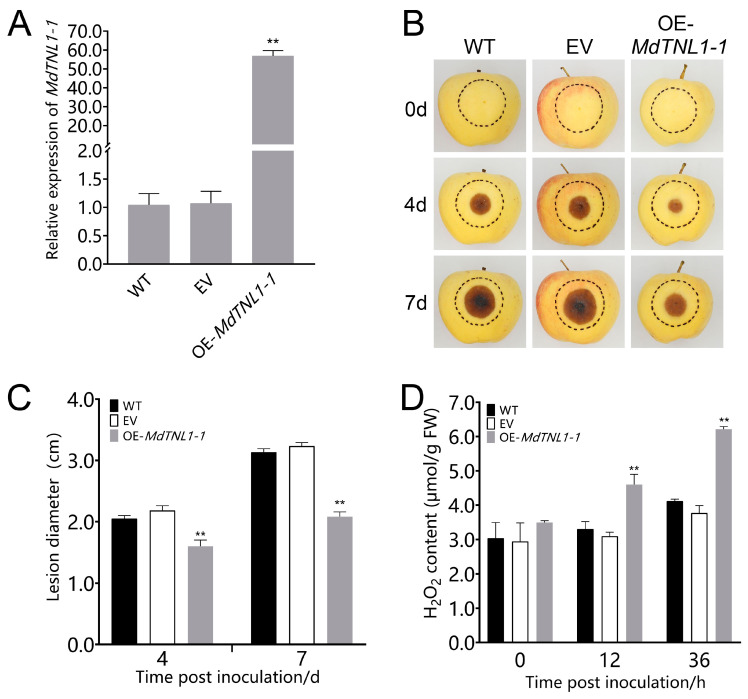
Effects of *MdTNL1-1* gene on the resistance of ‘Gala’ apple’s fruits to *C. fructicola*. (**A**) Expression of *MdTNL1-1* in the ‘Gala’ apple 9 days after infiltration. WT, wild type fruits with no treatment before inoculation. EV, infiltrated with *Agrobacterium* containing empty vectors, pCAMBIA1300; OE-*MdTNL1-1*, infiltrated with *Agrobacterium* harboring pCAMBIA1300-*MdTNL1-1*. Data are presented as the mean ± SD of three biological replicates. Asterisks indicate significant difference compared to WT (** *p* < 0.01, one-way ANOVA test by Tukey’s test). (**B**) Apple fruits were inoculated with *C. fructicola* 7 days after infiltration. Photographs were taken at 0, 4, and 7 dpi. (**C**) Apples’ lesion diameter measured at indicated time points. (**D**) H_2_O_2_ accumulation analyzed at 36 hpi. Data on (**C**,**D**) are presented as the mean ± SD of three biological replicates. Different asterisks indicate a significant difference (** *p* < 0.01, two-way ANOVA followed by Tukey’s post hoc test). Asterisks indicate significant difference compared to WT in the same hours.

**Figure 5:**
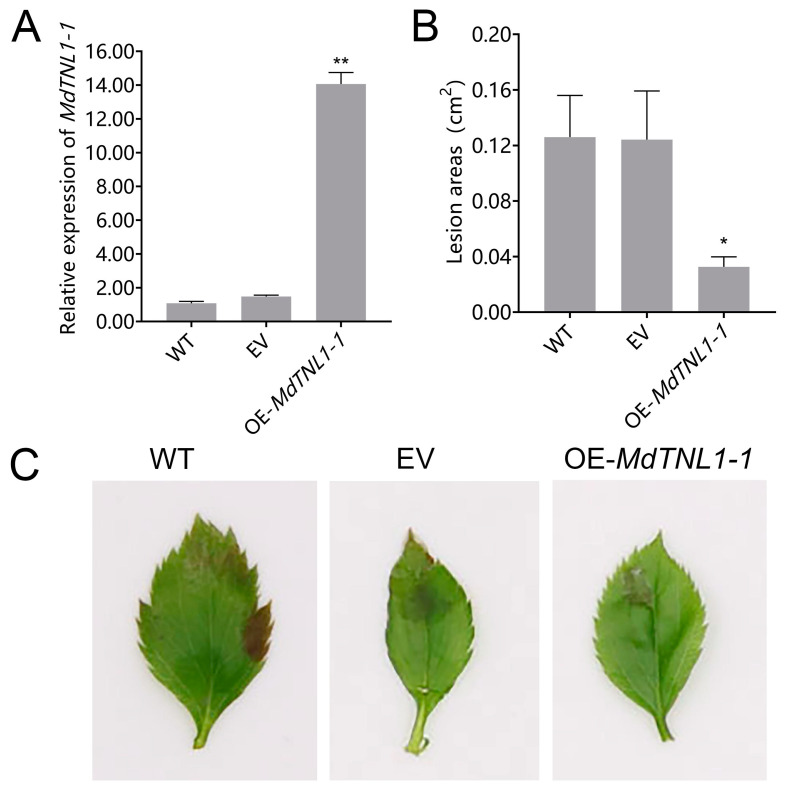
Effects of *MdTNL1-1* gene on the resistance of ‘Gala 3′ leaves to *C. fructicola.* (**A**) *MdTNL1-1* transcript levels of leaves from ‘Gala 3′ overexpressing *MdTNL1-1* or empty vector (EV: pCAMBIA1300) and WT plant 4 days after *C. fructicola* infection. (**B**) Statistics of lesion areas in leaves 3 days after inoculation with *C. fructicola* in transient expressed leaves. (**C**) The phenotypes of leaves from transient expressed leaves 4 days after *C. fructicola* infection. Data (A,B) are presented as means ± SD of three biological replicates. Different asterisks indicate a significant difference. Asterisk indicates significant difference compared to WT (* *p* < 0.05, ** *p* < 0.01; one-way ANOVA test by Tukey’s test).

**Figure 6:**
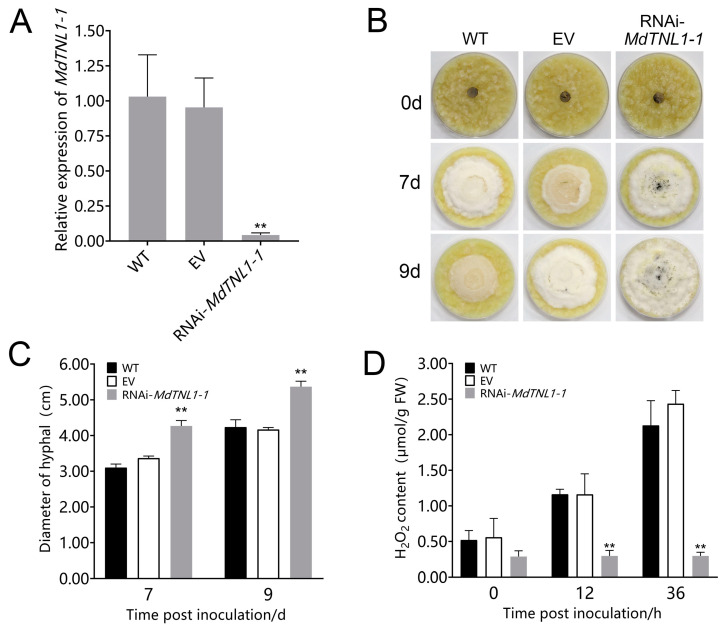
Effects of *MdTNL1-1* silencing on the ‘Orin’ calli to *C. fructicola*. (**A**) *MdTNL1-1* gene expressed in apple calli detected by qRT-PCR. EV, the calli transformed with PK7GWIW2D, an empty used here for control. Data are presented as the mean ± SD of three biological replicates. Asterisks indicate significant differences compared to WT (** *p* < 0.01; one-way ANOVA test by Tukey’s test). (**B**) Effects of *MdTNL1-1* silencing on the calli resistance. The photos were taken 0, 7, or 9 days after inoculation with *C. fructicola*. (**C**) Quantitation of *C. fructicola growth* on apple calli. (**D**) H_2_O_2_ accumulation of ‘Orin’ calli. Data on (C,D) are presented as the mean ± SD of three biological replicates. Different asterisks indicate a significant difference (** *p* < 0.01; two-way ANOVA followed by Tukey’s post hoc test). Asterisks indicate significant difference compared to WT in the same hours.

To investigate whether *MdTNL1-1* was involved in the resistance to GLS, overexpression was performed in leaves and fruits of susceptible ‘Gala’ using the agroinfiltration method. Quantitative RT-PCR showed that the significantly increased expressions of *MdTNL1-1* were observed in leaves and fruits subjected to gene overexpression (Figure 4 and Figure 5, respectively). Overexpression of *MdTNL1-1* improved the resistance of apple leaves to *C. fructicola* (Figure 5B,C). The similar phenomenon was also observed in fruits. That is, overexpression of *MdTNL1-1* enhanced fruit resistance to *C. fructicola* (Figure 4B,C). Conversely, overexpression of *MdTNL1-7* in fruits enhanced pathogen infection (Figure S2). These results indicate that *MdTNL1-1* plays a positive role in GLS resistance in apple. In order to investigate whether *MdTNL1-1* expression affects the growth of *C. fructicola*, we further knocked down the expression of *MdTNL1-1* in ‘Orin’ calli using RNAi technique. Compared with wild type (WT) and empty vector (EV: pCAMBIA1300), *MdTNL1-1*-silencing calli exhibited significantly reduced *MdTNL1-1* expression and increased the growth of the pathogenic fungi (Figure 6). These results revealed that *MdTNL1-1* expression affected the immune response capacity of apple to *C. fructicola*.

### 2.5. MdTNL1-1 Influenced the Expression of PR Genes, and SA and JA Signaling

**Figure 7:**
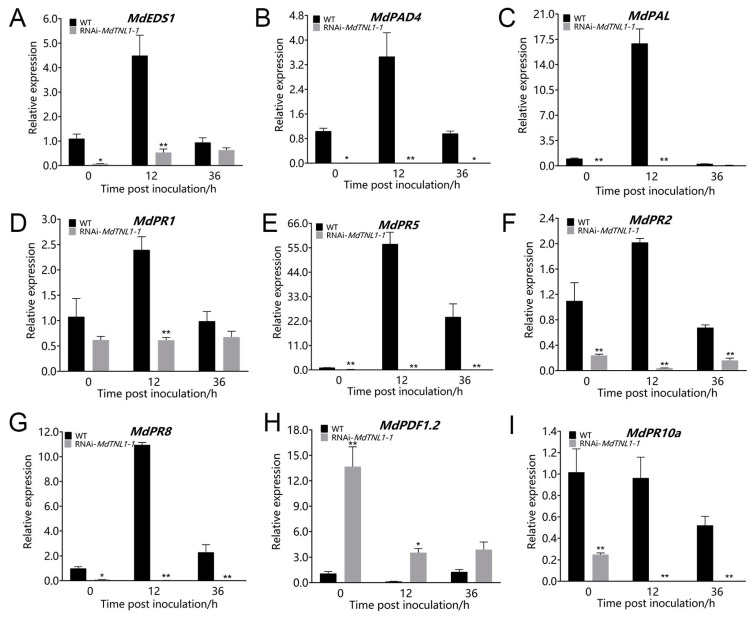
Effects of *MdTNL1-1*-silencing on the expression of PR genes and SA-pathway- and JA-pathway-related genes. (**A**–**I**) The relative expression of *MdEDS1*, *MdPAD4*, *MdPAL*, *MdPR1*, *MdPR5*, *MdPR2*, *MdPR8*, *MdPDF1.2*, and *MdPR10a* genes, respectively, in calli 0, 12, or 36 h after inoculation with *C. fructicola*. Different asterisks indicate a significant difference (* *p* < 0.05, ** *p* < 0.01; two-way ANOVA followed by Tukey’s post hoc test). Asterisks indicate significant difference compared to WT in the same hours.

To determine whether *MdTNL1-1* influences the defense responses, we investigated the expression of PR genes, *MdEDS1*, *MdPAD4*, *MdPAL*, and *MdPDF1.2. MdEDS1*, *MdPAD4*, and *MdPAL* are important for SA synthesis [22,23]. *MdPR1* and *MdPR5* are involved in SA signaling pathway [24]. *MdPDF1.2* is involved in JA signaling pathway [25]. *MdTNL1-1*-silencing calli were sampled at 0, 12, and 36 h after inoculation with *C. fructicola.* Quantitative RT-PCR revealed that *MdEDS1*, *MdPAD4*, *MdPAL*, *MdPR1*, *MdPR5*, *MdPR2*, and *MdPR8* showed enhanced expression in WT calli after inoculation with *C. fructicola* (Figure 7). However, in *MdTNL1-1-*silencing calli, these genes exhibited lower expression compared with that in control calli. Moreover, the higher *MdPDF1.2* expression was observed in *MdTNL1-1*-silencing calli than that in control calli after inoculation. *MdPR10a* expression did not show significant difference between WT and *MdTNL1-1*-silencing calli after inoculation. The above results indicate that *C. fructicola* infection influences SA and JA signaling as well as PR gene expression, suggesting that *MdTNL1-1* contributes to resistance to *C. fructicola* by affecting SA and JA signaling pathways. 

## 3. Discussion

In this work, we screened SNP loci using KASP technology in resistant and susceptible apple populations and obtained the GLS resistance associated SNP_7309212_ in this study. The SNP_7309212_ is located at the coding sequence of *MdTNL1* gene in chromosome 15 (Figure 1; Tables S4 and S5). *MdTNL1* gene expression was higher in the leaves of resistant cultivar after *C. fructicola* infection than that in susceptible one, and the expression of the *MdTNL1* gene was induced by *C. fructicola* (Figure S2B). KASP, HRM, and PCR sequencing revealed SNP_7309212_ difference between resistant and susceptible apple cultivars (Figure 1C,D; Tables S3–S5). SNP_7309212_ in the parents ‘Fuji’ and ‘Golden delicious’ showed AA and AT, respectively, whereas in the hybrid F1, homozygous AA only existed in the resistant apple, while heterozygous AT and homozygous TT were only present in the susceptible apple varieties, which is consistent with previous reports that GLS resistance was controlled by recessive single gene [3,21]; thus, we clearly found a strong association between the *MdTNL1* and GLS.

Allelic variation is an important driving force for plant co-evolution [26,27]. Previous research found that of rice *BPH* gene responsible for the resistance to BPH organism includes eight alleles [17]. In a study of *Verticillium wilt* resistance of *GhDSC1* gene, it was confirmed that the SNP variation of TNL type gene led to the reduction of nucleotide binding probability, which in turn affected the different resistance of *G. hirsutum* to cotton verticillium wilt [28]. Similarly, we found seven isomers of *MdTNL1* (*MdTNL1-1*–*MdTNL1-7*) in apple, which showed polymorphisms in the LRR domain (Figure 2A). Therefore, polymorphisms in *MdTNL1* alleles are assumed to be important for apple defense against *C. fructicola*.

Most NLR genes play important roles in the process of plant disease resistance. They are important components of the plant immune system. A previous study showed that overexpression of NLR usually causes plant cell death [29]. Transient overexpression of *MdTNL1-1* in *N. benthamiana* [30] resulted in tissue necrosis and H_2_O_2_ accumulation (Figure 3C). Firstly, we found that *MdTNL1-1* gene triggered local resistance associated with programmed cell death. In addition, analysis of the tissue specificity of *MdTNL1-1* expression in apple by qRT-PCR indicated that *MdTNL1-1* functioned in leaves and fruits, two major organs infected by *C. fructicola* (Figure 3A). Finally, subcellular location using onion epidermal cells showed that the MdTNL1-1 protein is expressed in the nucleus and the cell membrane (Figure 3B). Therefore, the discovery of tissue necrosis and reactive oxygen species accumulation has a new understanding for our study of *MdTNL1-1* immune research.

## 4. Materials and Methods

### 4.1. Plant Materials and Growth Conditions

Fifty-six apple cultivars or lines, including 22 common cultivars and 34 F1 hybrid lines from a cross of ‘Golden Delicious’ with ‘Fuji’, were grown at the Jiaozhou Experimental Farm of Qingdao Agricultural University. The leaves from current-year shoots were used for resistance evaluation. Apple fruits (*Malus* × *domestica* cv ‘Gala3’) 120 days after full blossom (DAFB) were used for infiltration and inoculation. Tissue-cultured apple plants (*Malus domestica* cv ‘Gala3’) were grown on Murashige and Skoog (MS) medium containing 0.3 mg L^−1^ 6-benzylaminopurine (6-BA), 0.2 mg L^−1^ indole-3-acetic acid (IAA), and 0.1 mg L^−1^ gibberellins 3 (GA_3_) at 25 °C in a climate-controlled culture room with a 16/8 h light/dark photoperiod and sub-cultured every 4 weeks. Apple seedlings aged about 4 weeks old were and used for agro-infiltration and fungal infection experiments. 

### 4.2. Pathogen Inoculation and Resistance Evaluation

The GLS pathogen *C. fructicola,* kindly provided by Professor Baohua Li from Qingdao Agricultural University, was grown on potato dextrose agar (PDA) at 28 °C for 7 days. Then, *C. fructicola* was transferred to spore culture solution containing 10 g L^−1^ KNO_3_, 5 g L^−1^ KH_2_PO_4_, 2.5 g L^−1^ MgSO_4_·7H_2_O, 0.02 g L^−1^ Fe_2_(SO_4_)_3_, and 50 g sugar at 28 °C for 3–5 days. Its spores were collected and suspended in deionized water at a concentration of 1×10^5^ CFU mL^−1^. The detached leaves of 56 apple varieties or transiently expressed apple leaves or fruits were inoculated by dropping 10 μL conidia suspensions on the surface of leaves. After inoculation, the leaves were placed in Petri dishes and kept at 28 °C and 75% humidity. The detached leaves of ‘Fuji’ and ‘Golden Delicious’ collected from a field (5 cm in length) or tissue cultured apple ‘Gala3’ (1 cm in length) were sprayed with spore suspensions of *C. fructicola*. To evaluate the disease resistance ability of leaves, we defined four ranks. Leaves with lesion diameter between 0 and 2 mm belonged to high resistance, lesion diameter between 2 and 4 mm belonged to medium resistance, lesion diameter between 4 and 8 mm belonged to medium susceptibility, and lesion diameter of more than 8 mm belonged to high susceptibility.

### 4.3. DNA Extraction and HRM Analysis

Apple leaves from 56 apple cultivars or lines were collected for DNA extraction. DNA extraction was performed with a plant genomic DNA kit (Tiangen Biochemical Technology Co., Ltd, Beijing, China), following the manuals provided by manufacturer. The extracted DNA was used for PCR, KASP, and high-resolution melting analysis (HRM). The HRM was conducted with a LightCycler^®^480 High Resolution Melting Master Kit on a LightCycler^®^ 480 System (Roche, Switzerland). The reaction included 1 μL of genomic DNA (10 ng μL^−1^), 7.5 μL of 2 × Master Mix, 1.5 μL MgCl_2_ (2 mmol L^−1^), 0.5 μL of each primer (0.2 μmol L^−1^), and 4 μL of water. The program for PCR amplification was as follows: predenature at 95 °C for 10 min followed by 45 cycles of 95 °C for 15 s, 55 °C for 15 s, and 72 °C for 10 s. The HRM detection program for the amplified products was as follows: 95 °C for 1 min, 40 °C for 1 min, 65 °C for 1 s. The fluorescence information was collected at a frequency of 25 times °C^−1^ during the process of increasing 65 °C to 95 °C, and finally cooling down to 40 °C using the gene scanning software for HRM analysis of the LightCycler^®^480 system.

### 4.4. KASP Analysis 

Primers specific for SNP_s_ were designed for the PCR reaction using Primer 5. A gaaggtgaccaagttcatgct sequence linker was added to the 5’ end of primer A1 and a gaaggtcggagtcaacggatt sequence linker was added to the 5’end of primer A2. The final concentration of primers used was 10 μM, and the ratio of primer A1/primer A2/primer C was mixed in a volume ratio of 12:12:30. We followed LGC company KASP guidelines (https://doi.org/10.1016/j.isci.2021.102447, accessed on 4 May 2022). We set up a KASP reaction system of 10.14 μL, including 5 μL of 10 ng/μL DNA sample, 5 μL of 2 × master mix, and 0.14 μL mixed primers, taking care to set up a no template control (NTC) on each 96-well plate. The KASP reaction program was as follows: predenaturation phase 94 °C for 15 min; 10 cycles of denaturation (94 °C for 20 s) and renaturation extension (61–55 °C) phase setting with a decrease of 0.6 °C per cycle; and a final denaturation (94 °C for 20 s, 55 °C for 60 s) phase for 26 cycles. The resulting data were analyzed using Kraken (http://ccb.jhu.edu/software/kraken/MANUAL.html, accessed on 4 May 2022) software.

### 4.5. RNA Extraction and qRT-PCR

RNA extraction occurred from apple fruits, leaves of tissue-cultured plantlets, and calli. cDNA synthesis was performed according to the previously reported method [31]. Using GDR (https://www.rosaceae.org/, accessed on 4 May 2022) and FGENESH (http://www.softberry.com/, accessed on 4 May 2022), we cloned the sequence of the *MdTNL1* gene and obtained the target sequence by sequencing. Protein domain analysis was performed using SMART (http://smart.embl-heidelberg.de/, accessed on 4 May 2022). *MdTNL1* expression in ‘Fuji’ and ‘Golden Delicious’ was analyzed at 12, 24, 36, 48, and 72 h post-inoculation (hpi), and disease spots in the leaves were counted. The cDNA of susceptible leaves ‘Golden Delicious’ or resistant leaves ‘Fuji’ was used for PCR, and the PCR product was ligated into the pMD19-T vector and then was sequenced. qRT-PCR was performed using ChamQ SYBR Color qPCR Master Mix, according to the manuals (Vazyme, China). Relative expression of *MdTNL1-1* was calculated using the method of 2^−ΔΔCt^, and a *MdActin* gene was used as the internal reference [32,33]. The experiment was performed 3 times.

### 4.6. Cloning of MdTNL1

cDNA from the susceptible leaf ‘Golden Delicious’ or resistant leaves ‘Fuji’ was used as a template. The primers (named MdTNL1 forward or reserve) were used for PCR to clone *MdTNL1*. PCR amplification was performed with designed primers as previously described [34]. 

### 4.7. Transient Expression Assay in Tobacco Leaves, Apple Leaves, and Fruits

The CDS of *MdTNL1-1* was cloned and ligated into pCAMBIA1300 and PK7WIWG2D vectors to generate pCAMBIA1300-*MdTNL1-1*-*GFP* for overexpression and *MdTNL1-1*-PK7WIWG2D for RNA interference. According to the method previously described [35], we transformed the new constructed vector into *Agrobacterium* strain EHA105 and infiltrated *Agrobacteria* into tobacco leaves, leaves of ‘Gala3’ apple, and fruits of ‘Gala’ apple 120 DAFB. The leaves should be fully infiltrated, and the fruits were required to have an injection diameter of 5 cm. The tobacco leaves were visually examined and stained by trypan blue (TPB) or diaminobenzidine (DAB) 7 days after being injected [36,37]. *C. fructicola* was inoculated at the apple infiltration site 9 days after *Agrobacterium* infiltration with 0.5 μL. Resistance was assessed 4 days or 7 days after inoculation. Through our experimental observation, we found that in the early stage of *C. fruticola* infecting the host plant, although the pathogen destroyed the host plant, the host plant did not show disease in a short period of time, but the outbreak time of reactive oxygen species was rapid. Therefore, we selected the pathogen to evaluate the resistance after 4 and 7 days in fruit and 7 and 9 days in calli. The content of H_2_O_2_ was measured at 0, 12, and 36 h after inoculation using an H_2_O_2_ content detection kit (Solarbio Biotechnology Co., Ltd, Beijing, China) according to instructions provided by the manufacturer. *MdTNL1-1* expression analysis was performed 3 or 9 days after infiltration. 

### 4.8. Subcellular Localization of MdTNL1-1 and Genetic Transformation of Apple Calli

*Agrobacterium* EHA105 strain containing the MdTNL1-1-GFP fusion vector or the pCAMBIA1300-GFP control vector were infiltrated into onions. The infiltrated onions were incubated in the dark for 16–24 h, and then the cut samples were observed under a laser confocal microscope (Leica, Heidelberg, Germany) [38,39]. To silence *MdTNL1* in apple calli, 15-day-old calli were co-cultured with *Agrobacterium* carrying *MdTNL1-1*-PK7WIWG2D for two days on MS medium containing 1.5 mg L^−1^ 2,4-dichlorophenoxyacetic acid (2,4-D) and 0.225 mg L^−1^ at 25 °C. The calli were then transferred to a selective MS medium containing 200 mg L^−1^ cefotaxime and 100 mg L^−1^ kanamycin. The transgenic calli were inoculated with *C. fructicola* after 7 and 9 days to evaluate the disease resistance.

### 4.9. Expression Analysis of PR Genes and SA- and JA-Related Genes

The apple calli were treated with 1 × 10^5^ CFU ml^−1^ spore suspension, and the calli samples were collected at 12 h and 36 h after treatment for RT-qPCR. Genes expression analysis of *MdEDS1*, *MdPAD4*, *MdPAL*, *MdPR1*, *MdPR5*, *MdPR2*, *MdPR8*, *MdPDF1.2*, and *MdPR10a* were performed using RT-qPCR, as previously described with 4.5 part. The experiment was performed by using three independent biological replicates, and the significant difference was analyzed by two-way ANOVA test (Figure 7).

## 5. Conclusions

TNL family genes are important objects for studying plant immune defense. Through this study in selected 17 SNP sites within the GLS mapping interval, we found SNP_7309212_ marker in *MdTNL1* was closely associated with GLS resistance. The expression of *MdTNL1* was significantly improved in response to specific pathogenic bacteria. Importantly, we showed that *MdTNL1-1* overexpression improved resistance of apple to *C. fructicola*. *MdTNL1-1* can play a defensive role against GLS by enhancing ROS capacity as well as regulating JA and SA signaling pathway genes and PR gene expression. Overall, the perspective presented in this study may open a new research path for understanding the complex mechanisms of resistance to GLS in apple plants.

## Data Availability

The authors declare that all data and materials supporting the findings of this study are available within the article. The data that support the findings of this study are available from the corresponding author upon reasonable request.

## Supplementary Materials

The following supporting information can be downloaded at: https://www.mdpi.com/article/10.3390/ijms23116323/s1.
