# Supplementary material for: A TIR-NBS-LRR Gene MdTNL1 Regulates Resistance to Glomerella Leaf Spot in Apple
Source: Int J Mol Sci. 2022 Jun 5;23(11):6323. doi: 10.3390/ijms23116323 (PMC9181576; doi:10.3390/ijms23116323)
Supplement: Supplementary file 1 [file ijms-23-06323-s001.zip › ijms-1735360-supplementary.pdf]

Supplementary Materials

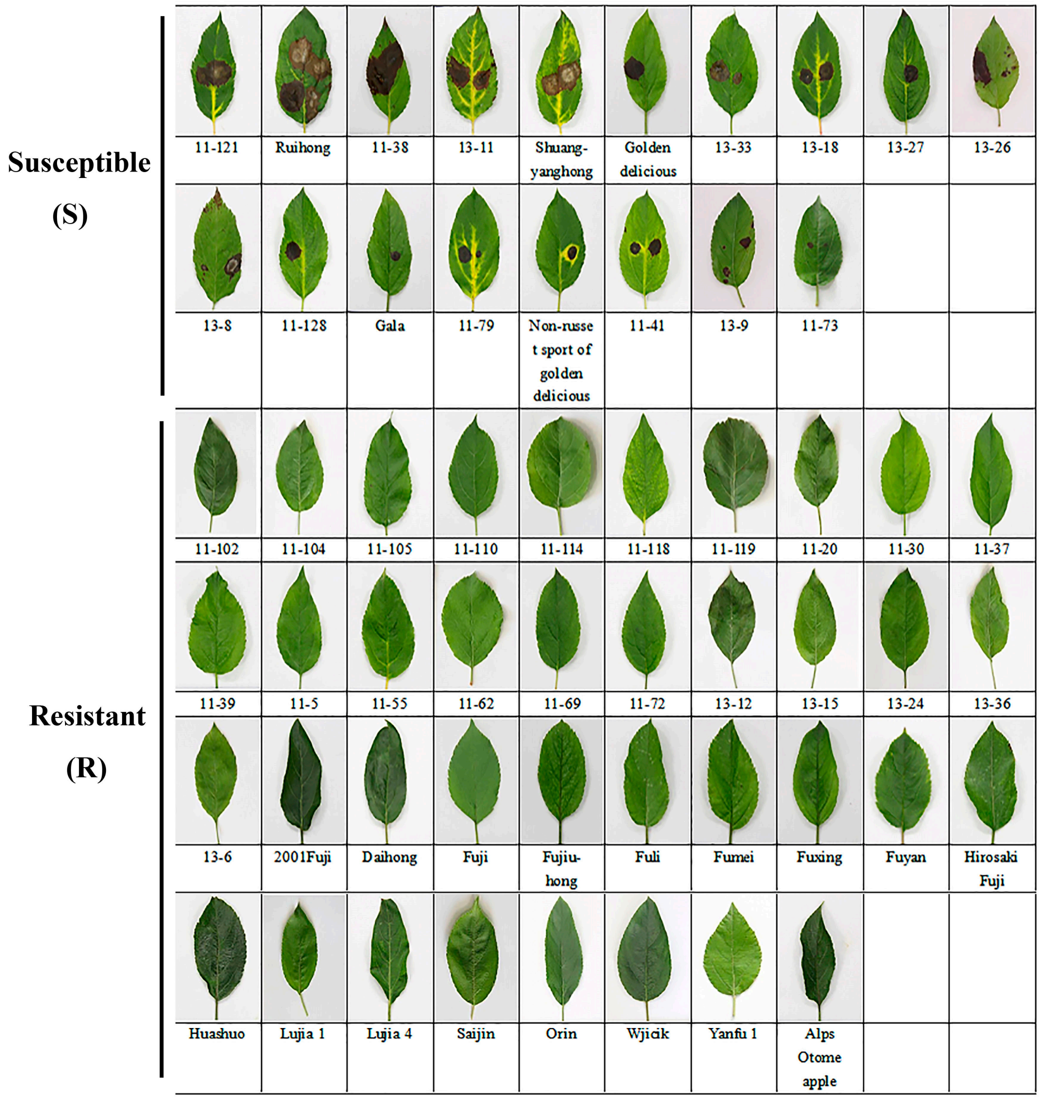

Figure S1. Disease resistance phenotype of different apple leaves to *C. fructicola*.

Table S1. Phenotypic identification of GLS resistance in apple populations.

Number	Cultivars	Lesion diameter of single leaf（cm）	Phenotype
1	11-121	3	Susceptible (S)
2	Ruihong	2.9	Susceptible (S)
3	11-38	2.4	Susceptible (S)
4	13-11	2.22	Susceptible (S)
5	Shuangyanghong	1.6	Susceptible (S)
6	Golden delicious	1.4	Susceptible (S)
7	13-33	1.33	Susceptible (S)
8	13-18	1.17	Susceptible (S)
9	13-27	1.1	Susceptible (S)
10	13-26	1	Susceptible (S)
11	13-8	1	Susceptible (S)
12	11-128	0.9	Susceptible (S)

13	Gala	0.8	Susceptible (S)
14	11-79	0.76	Susceptible (S)
15	Non-russet sport of golden delicious	0.7	Susceptible (S)
16	11-41	0.67	Susceptible (S)
17	13-9	0.5	Susceptible (S)
18	11-73	0.5	Susceptible (S)
19	11-102	0	Resistant (R)
20	11-104	0	Resistant (R)
21	11-105	0	Resistant (R)
22	11-110	0	Resistant (R)
23	11-114	0	Resistant (R)
24	11-118	0	Resistant (R)
25	11-119	0	Resistant (R)
26	11-20	0	Resistant (R)
27	11-30	0	Resistant (R)
28	11-37	0	Resistant (R)
29	11-39	0	Resistant (R)
30	11-5	0	Resistant (R)
31	11-55	0	Resistant (R)
32	11-62	0	Resistant (R)
33	11-69	0	Resistant (R)
34	11-72	0	Resistant (R)
35	13-12	0	Resistant (R)
36	13-15	0	Resistant (R)
37	13-24	0	Resistant (R)
38	13-36	0	Resistant (R)
39	13-6	0	Resistant (R)
40	2001Fuji	0	Resistant (R)
41	Daihong	0	Resistant (R)
42	Fuji	0	Resistant (R)
43	Fujiuhong	0	Resistant (R)
44	Fuli	0	Resistant (R)
45	Fumei	0	Resistant (R)
46	Fuxing	0	Resistant (R)
47	Fuyan	0	Resistant (R)
48	Hirosaki Fuji	0	Resistant (R)
49	Huashuo	0	Resistant (R)
50	Lujia 1	0	Resistant (R)
51	Lujia 4	0	Resistant (R)
52	Saijin	0	Resistant (R)
53	Orin	0	Resistant (R)
54	Wjicik	0	Resistant (R)
55	Yanfu 1	0	Resistant (R)
56	Alps Otome apple	0	Resistant (R)

Table S2. 17 SNP sites associated with GLS resistance in apple

Number	SNP sites	Gene ID	Chromosome	nt change	aa change
1	$SNP_{5360708}$	MD15G1078500	15	A/G	Met/Val
2	$SNP_{5450718}$	MD15G1080100	15	G/A	Gln/*
3	$SNP_{6282372}$	MD15G1090600	15	A/T	Cys/Ser

4	SNP$_{7298678}$	MD15G1103600	15	G/A	Ser/Leu
5	SNP$_{7298811}$	MD15G1103600	15	C/G	Glu/Gln
6	SNP$_{7298886}$	MD15G1103600	15	T/G	Ile/Leu
7	SNP$_{7302373}$	MD15G1103600	15	T/A	Glu/Asp
8	SNP$_{7306449}$	MD15G1103700	15	A/C	Glu/Ala
9	SNP$_{7308234}$	MD15G1103700	15	G/A	Asp/Asn
10	SNP$_{7309015}$	MD15G1103800	15	G/C	Arg/Thr
11	SNP$_{7309020}$	MD15G1103800	15	G/A	Glu/Lys
12	SNP$_{7309212}$	MD15G1103800	15	A/T	Ile/Phe
13	SNP$_{7391379}$	MD15G1105700	15	G/T	Ser/Arg
14	SNP$_{7401464}$	MD15G1106000	15	T/A	Thr/Ser
15	SNP$_{7479500}$	MD15G1106800	15	A/T	Asn/Ile
16	SNP$_{7479643}$	MD15G1106800	15	T/C	Trp/Arg
17	SNP$_{7479710}$	MD15G1106800	15	T/G	Ile/Arg

Table S3. Genotype of other 16 SNP sites in hybridization F1 progenies from 'Golden Delicious' × 'Fuji' and field cultivar populations

*DNA samples	SNP$_{7391379}$ (G/T)	*DNA samples	SNP$_{5360708}$ (A/G)	*DNA samples	SNP$_{7298811}$ (C/G)	*DNA samples	SNP$_{7298886}$ (T/G)
R11-33	GG	R11-52	GG	R11-101	CC	R11-101	GT
R11-72	GT	R11-122	GG	R11-114	CC	R11-104	TT
R13-12	GG	R11-36	GG	S11-120	CC	R11-114	TT
R13-29	TT	R11-45	GG	S11-41	CC	R Changfu 2	TT
R Saijin	GG	R11-8	GG				
S11-115	TT	R13-29	AA				
S11-121	TT	R13-36	GG				
S13-33	TT						
S13-7	TT						
S13-8	TT						
*DNA samples	SNP$_{5450718}$ (G/A)	*DNA samples	SNP$_{7298678}$ (G/A)	*DNA samples	SNP$_{7479500}$ (A/T)	*DNA samples	SNP$_{7479643}$ (T/C)
R11-52	GA	R11-101	GG	R11-105	TT	R11-126	TT
R11-122	AA	R11-104	GG	R11-126	TT	S13-13	CT
R11-36	AA	R11-114	GG	R13-15	TT	S13-27	CT
R11-45	GA	RChangfu 2	GG	S13-27	AT	S13-31	CT
R11-8	GA	S11-120	GG	S13-7	AT	S Ruihong	CT
R13-29	GG	S11-41	GG	S Ruihong	AT	S Shuang-yanghong	CT
*DNA samples	SNP$_{7306449}$ (A/C)	*DNA samples	SNP$_{7308234}$ (G/A)	*DNA samples	SNP$_{7479710}$ (T/G)	*DNA samples	SNP$_{6282372}$ (A/T)
R11-102	AA	R11-102	GG	R11-105	GG	R11-52	TT
R11-106	AA	R11-106	GG	R11-106	GG	R11-106	TT
R11-107	AA	R11-107	GG	R11-118	GG	R11-110	TT
R11-12	AA	R11-12	GG	R11-126	GG	R11-122	TT
R11-20	AC	R11-20	AG	R11-30	TT	R11-33	AT
R11-70	AA	R11-70	GG	R11-37	TT	R11-36	TT
S11-121	CC	S11-121	AA	R Saijin	TT	R11-37	AT
S13-26	CC	S13-26	AA	S11-27	TT	R11-45	TT
S13-34	AC	S13-34	AA	S13-11	TT	R11-55	AT
S13-8	CC	S13-8	AA	S13-13	TT	R11-69	TT
S Golden deli-cious	CC			S13-27	TT	R11-8	TT

				S13-31	TT	R13-12	TT
				S13-33	TG	R13-35	TT
				S13-7	TG	R13-36	TT
				S13-9	TG	R13-6	TT
				S Golden delicious	TT	S11-121	AT
				S Ruihong	TT	S11-38	AT
						S11-41	AT
						S11-86	AT
						S13-11	AA
						S13-4	AT
						S13-5	AT
						S13-9	AT
						S Shuangyanghong	AT

*DNA samples	SNP$_{7302373}$ (T/A)	*DNA samples	SNP$_{7309015}$ (G/C)	*DNA samples	SNP$_{7309020}$ (G/A)	*DNA samples	SNP$_{7401464}$ (T/A)
R11-101	TT	R11-101	GG	R11-101	GA	R11-101	TA
R11-102	TT	R11-102	GG	R11-102	GA	R11-102	TA
R11-105	TT	R11-105	GC	R11-105	GA	R11-104	TA
R11-107	TT	R11-110	GG	R11-106	GA	R11-105	TA
R11-118	TT	R11-118	GC	R11-107	GA	R11-106	TA
R11-12	TT	R11-119	GG	R11-110	GA	R11-107	TA
R11-20	TT	R11-12	GG	R11-118	GA	R11-110	TA
R11-33	TT	R11-126	GC	R11-119	GA	R11-114	TA
R11-37	TA	R11-20	GG	R11-122	GA	R11-118	TA
R11-45	AA	R11-30	GG	R11-126	GA	R11-119	TA
R11-52	TT	R11-33	GC	R11-30	GA	R11-122	TA
R11-70	TT	R11-37	GG	R11-33	GA	R11-126	TA
R Fuli	TA	R11-45	GC	R11-36	GA	R11-20	TA
S11-115	TA	R11-49	GG	R11-37	GA	R11-30	TA
S11-121	TT	R11-5	GG	R11-39	GA	R11-33	TA
S11-129	TA	R11-52	GG	R11-45	GA	R11-36	TA
S11-27	TT	R11-55	GG	R11-49	GA	R11-37	TA
S11-38	TT	R11-62	GG	R11-5	GA	R11-39	TT
S11-41	TT	R11-69	GG	R11-52	GA	R11-45	TA
S11-86	TA	R11-72	GG	R11-55	GA	R11-5	TA
S13-26	TA	R13-15	GG	R11-69	GA	R11-52	TA
S13-33	TA	R13-20	GG	R11-70	GA	R11-55	TA
S13-34	TT	R13-23	GG	R11-72	GA	R11-62	TT
S13-5	TA	R13-24	GC	R13-12	GA	R11-69	TA
S13-8	TT	R13-35	GG	R13-20	GA	R11-70	TA
S13-9	AA	R13-36	GC	R13-23	GA	R11-72	TA
S Golden delicious	TT	R13-6	GG	R13-24	GA	R11-8	TA
		R 2001Fuji	GC	R13-29	GA	R13-12	TA
		R Fuli	GG	R13-36	GA	R13-15	TA
		R Fumei	GC	R 2001Fuji	GA	R13-20	TT
		R Fuxing	GG	R Fuli	GA	R13-23	TA
		R Fuyan	GC	R Fumei	GA	R13-24	TA
		R Guoguang	GC	R Changfu 2	GA	R13-29	TA
		R Hirosaki Fuji	GC	R Fuxing	GA	R13-36	TA

R Huashuai	GC	R Fuyan	GA	R 2001Fuji	TA
R Huashuo	GC	R Guoguang	GA	R Daihong	TA
R Hongxun 1	GC	R Hirosaki Fuji	GA	RFumei	TA
R Lujia 1	GG	R Huashuai	GA	R Changfu 2	TA
R Lujia 4	GG	R Huashuo	GA	R Fuxing	TA
R Wjicik	GC	R Hongxun 1	GA	R Fuyan	TA
R Xinhongxing	GC	R Lujia 1	GA	R Guoguang	TA
R Alps Otome apple	GC	R Lujia 4	GA	R Hirosaki Fuji	TA
R Fujiuhong	GC	R Saijin	GA	R Huashuai	TA
S11-113	GC	R Wjicik	GA	R Huashuo	TA
S11-115	GC	R Fujiuhong	GA	R Hongxun 1	TA
S11-120	GC	R Xinhongxing	GA	R Lujia 1	TA
S11-121	GC	R Alps Otome apple	GA	R Lujia 4	TA
S11-128	GC	S11-113	GA	R Saijin	TA
S11-129	GC	S11-115	GA	R Wjicik	TA
S11-14	GC	S11-117	GA	R Fujiuhong	TA
S11-27	GC	S11-120	GA	R Xinhongxing	TA
S11-38	GC	S11-121	GA	R Alps Otome apple	TA
S11-41	GC	S11-128	GA	S11-113	TA
S11-79	GC	S11-129	GA	S11-117	TA
S11-86	GC	S11-14	GA	S11-120	TA
S12-27	GG	S11-27	GA	S11-128	TA
S12-32	GC	S11-38	GA	S11-129	TA
S12-70	GG	S11-41	GA	S11-14	TA
S13-11	GC	S11-79	GA	S11-38	TA
S13-13	GC	S11-86	GA	S11-41	TA
S13-18	GC	S12-27	GG	S11-73	TA
S13-26	GG	S12-32	GA	S11-79	TA
S13-27	GC	S12-70	GA	S11-86	TA
S13-28	GC	S13-11	GA	S12-27	TA
S13-31	GC	S13-13	GA	S12-32	TA
S13-33	GC	S13-18	GA	S12-70	TA
S13-34	GC	S13-26	GA	S13-11	TA
S13-5	GC	S13-27	GA	S13-13	TA
S13-7	GC	S13-28	GA	S13-18	TA
S13-9	GG	S13-31	GA	S13-27	TA
S Gala	GC	S13-33	GA	S13-28	TA
S Golden delicious	GG	S13-34	GA	S13-31	TA
S Ruihong	GG	S13-5	GA	S13-5	TA
S Shuangyanghong	GC	S13-7	GA	S13-7	TA
S Non-russet sport of golden delicious	GC	S13-8	GA	S13-9	TA
		S13-9	GA	S Gala	TA
		S Gala	GA	S Golden delicious	TA

S Golden delicious	GA	S Shuang-yanghong	AA
S Shuang-yanghong	GA	S Non-russet sport of golden delicious	TA
S Non-russet sport of golden delicious	GA		

*Adding ‘R’ or ‘S’ before the name of DNA sample indicates that the sample material is ‘resistant’ or ‘susceptible’, and the number after ‘R’ or ‘S’ is the strain code.

Table S4. Genotype of $SNP_{7309212}$ in hybridization F1 progenies from ‘Golden Delicious’ × ‘Fuji’ and field cultivar populations

Number	DNA samples	Phenotype	$SNP_{7309212}$ (A/T)
1	11-102	R	AA
2	11-104	R	AA
3	11-105	R	AA
4	11-110	R	AA
5	11-114	R	AA
6	11-119	R	AA
7	11-20	R	AA
8	11-30	R	AA
9	11-37	R	AA
10	11-39	R	AA
11	11-5	R	AA
12	11-55	R	AA
13	11-62	R	AA
14	11-69	R	AA
15	13-12	R	AA
16	13-15	R	AA
17	13-2413-8	R	AA
18	13-36	R	AA
19	13-6	R	AA
20	Fuji	R	AA
21	Daihong	R	AA
22	Fujiuhong	R	AA
23	Fuli	R	AA
24	Hirosaki Fuji	R	AA
25	Huashuo	R	AA
26	Lujia 1	R	AA
27	Saijinn	R	AA
28	Orin	R	AA
29	Wjicik	R	AA
30	Alps Otome apple	R	AA
31	11-121	S	AT
32	11-128	S	AT
33	11-73	S	AT
34	11-79	S	AT
35	13-18	S	AT

36	13-33	S	AT
37	13-8	S	AT
38	Gala	S	AT
39	Golden delicious	S	AT
40	Ruihong	S	AT
41	Shuangyanghong	S	AT
42	Non-russet sport of golden delicious	S	AT

Table S5. Genotypes of apple cultivars with different resistance at $SNP_{7309212}$ locus

Number	DNA smaples	Phenotype	$SNP_{7309212}$ （A/T）	Sequence peak of $SNP_{7309212}$	Num-ber	DNA smaples	Pheno-type	$SNP_{7309212}$ （A/T）	Sequence peak of $SNP_{7309212}$
1	11-102	R	AA	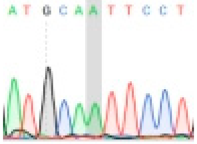	29	Fuyan	R	AA	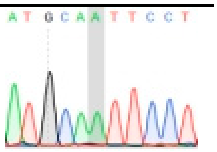
2	11-104	R	AA	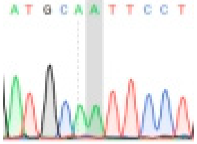	30	Hirosaki Fuji	R	AA	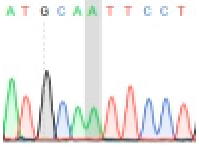
3	11-105	R	AA	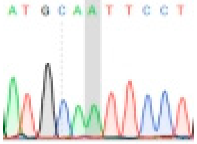	31	Huashuo	R	AA	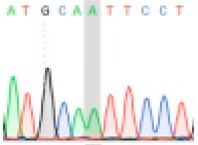
4	11-110	R	AA	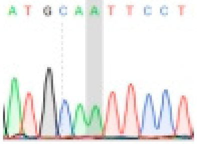	32	Lujia 4	R	AA	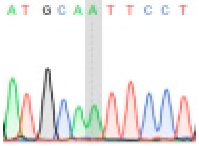
5	11-114	R	AA	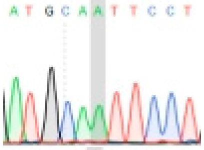	33	Saijin	R	AA	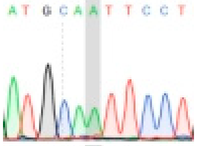
6	11-118	R	AA	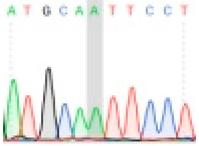	34	Orin	R	AA	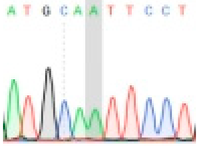
7	11-119	R	AA	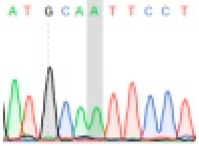	35	Wjicik	R	AA	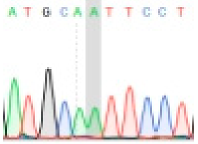
8	11-20	R	AA	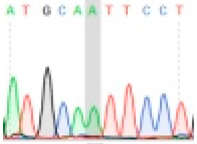	36	Yanfu 1	R	AA	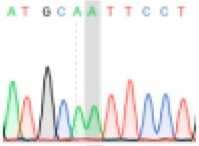
9	11-30	R	AA	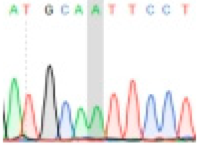	37	Alps Otome apple	R	AA	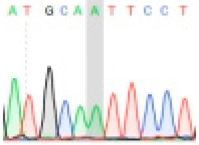

10	11-37	R	AA	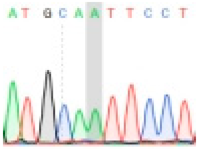	38	11-121	S	AT	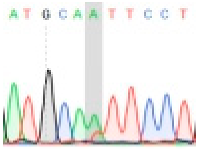
11	11-39	R	AA	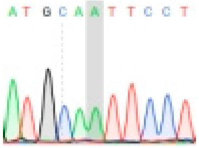	39	11-128	S	AT	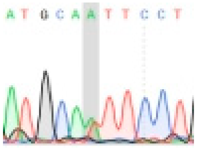
12	11-5	R	AA	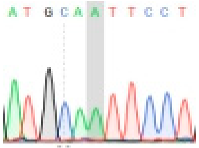	40	11-38	S	AT	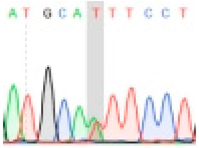
13	11-55	R	AA	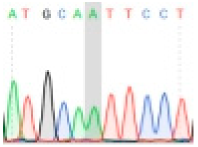	41	11-41	S	AT	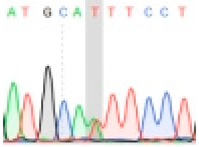
14	11-62	R	AA	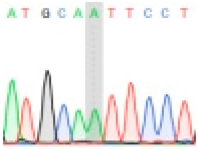	42	11-73	S	AT	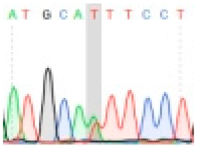
15	11-69	R	AA	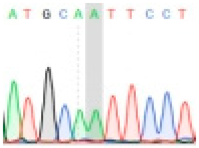	43	11-79	S	AT	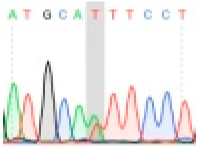
16	11-72	R	AA	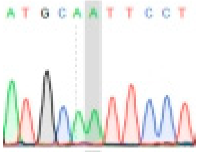	44	13-11	S	AT	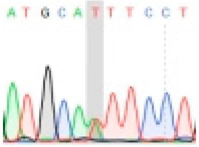
17	13-12	R	AA	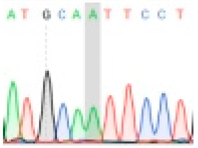	45	13-18	S	AT	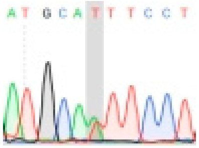
18	13-15	R	AA	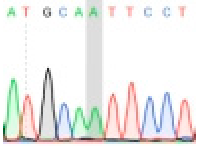	46	13-26	S	AT	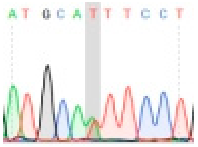
19	13-24	R	AA	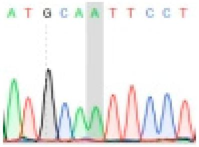	47	13-27	S	AT	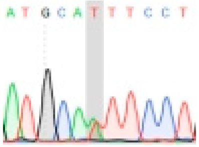
20	13-36	R	AA	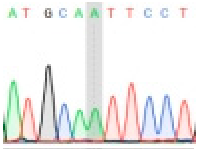	48	13-33	S	AT	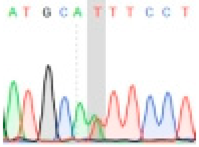
21	13-6	R	AA	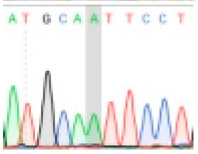	49	13-8	S	AT	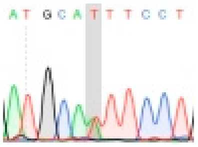

22	2001Fuji	R	AA	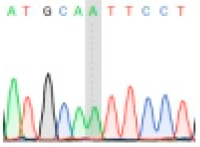	50	13-9	S	AT	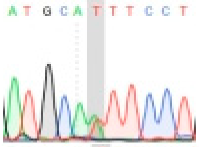
23	Fuji	R	AA	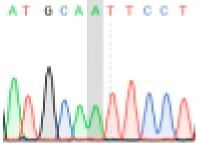	51	Gala	S	AT	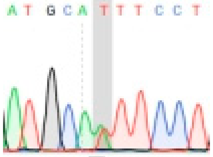
24	Dai-hong	R	AA	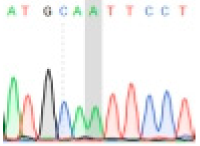	52	Golden delicious	S	AT	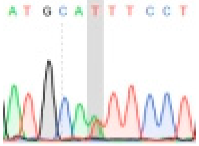
25	Fu-jiuhong	R	AA	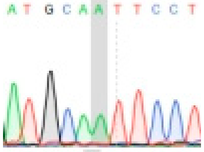	53	Ruihong	S	AT	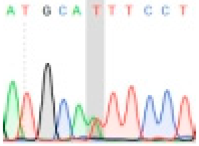
26	Fuili	R	AA	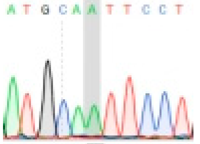	54	Shuang-yanghong	S	AT	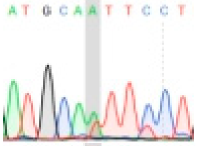
27	Fumei	R	AA	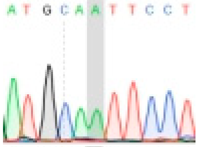	55	Non-rus-set sport of golden delicious	S	AT	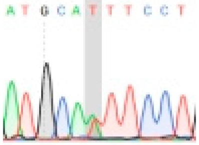
28	Fuxing	R	AA	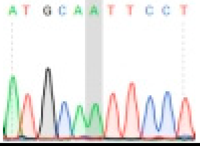					

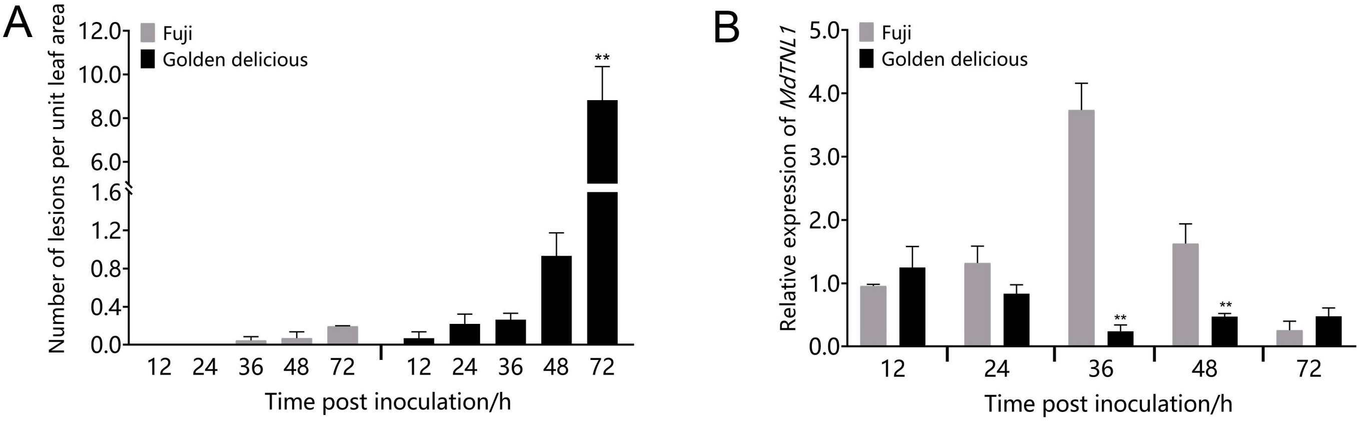

Figure S2. Effects of *MdTNL1* gene on the resistance of 'Fuji' and 'Golden delicious' apple's leaves to *C. fructicola*. (A) The number of disease spots per unit leaf area. (B) The expression of *MdTNL1* in leaves of 'Fuji' and 'Golden delicious' after inoculated with *C. fructicola*. Data are presented as the mean ± SD of three biological replicates (** $p < 0.01$, two-way ANOVA followed by Tukey's post hoc test). Asterisks in Fig S2A indicate significant difference compared to 12 hours in the same group and asterisks in Fig S2B indicate significant difference compared to 'Fuji' in the same hours.

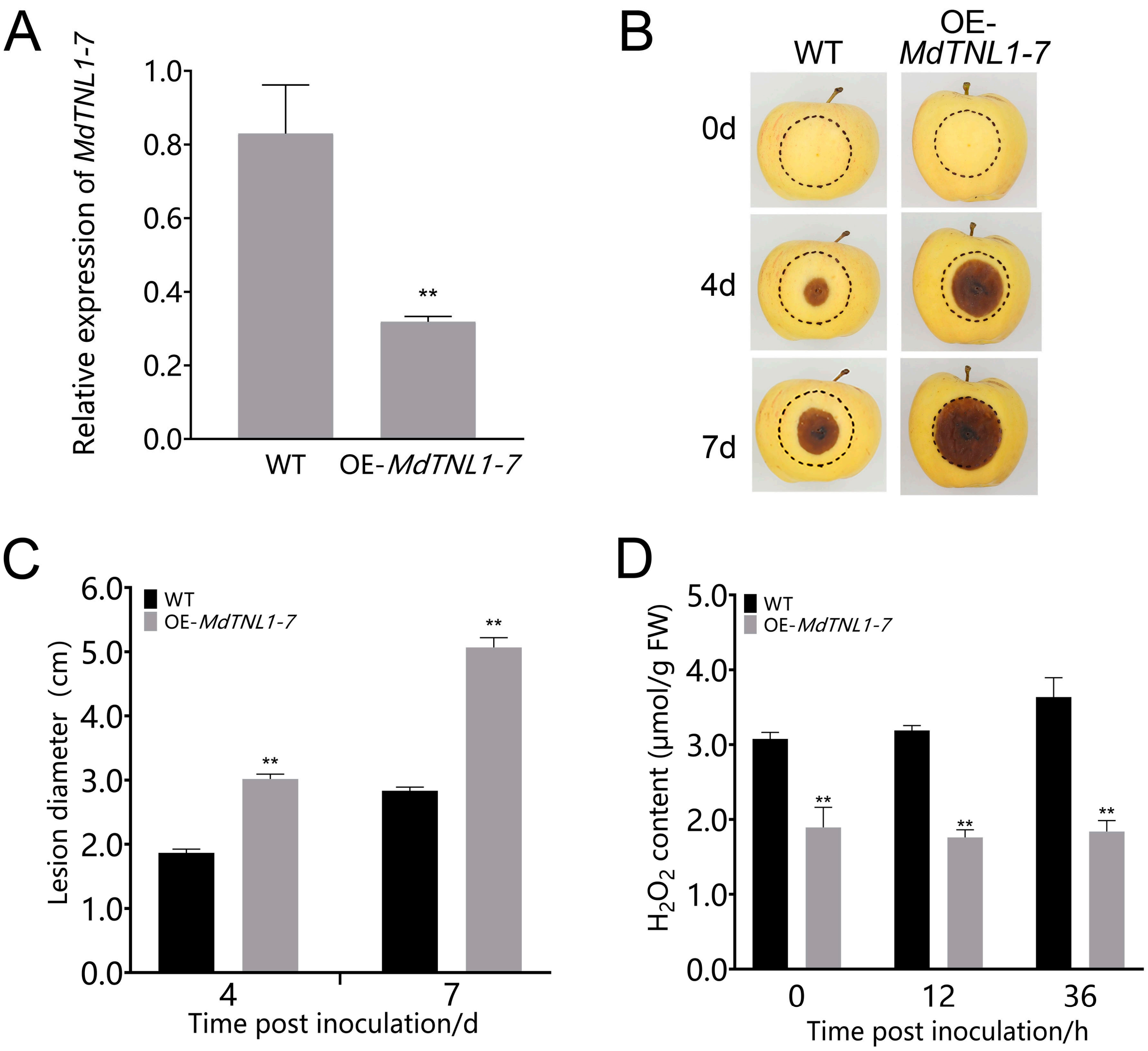

Figure S3. Effects of *MdTNL1-7* gene on the resistance of 'Gala' apple's fruits to *C. fructicola*. (**A**) Expression of *MdTNL1-7* in the 'Gala' apple 9 days after infiltration. Data are presented as the mean ± SD of three biological replicates. (; ** $p < 0.01$, one-way ANOVA test by Tukey's test). (**B**) Apple fruits were inoculated with *C. fructicola* 7 days after infiltration. Photographs were taken at 0, 4, and 7 dpi. (**C**) Apples' lesion diameter measured at indicated time points. (**D**) H_2O_2 accumulation analyzed at 36 hpi. Data on (C) and (D) are presented as the mean ± SD of three biological replicates. Different asterisks indicate a significant difference (* $p < 0.05$; ** $p < 0.01$, two-way ANOVA followed by Tukey's *post hoc* test). Asterisks indicate significant difference compared to WT in the same hours.

Table S6. Gene-specific primers used for PCR and qRT-PCR.

Gene name	Primer	Sequence 5'-3'
MdTNL1OE1	Forward	TTGATTACTGTGGAGGGTTG
MdTNL1OE1	Reserve	TCATTTCTGCCTTCCTTGTA
MdTNL1	Forward	ATGGATACTACCATCACAGC
MdTNL1	Reserve	TCAACTGATTCCGAATTTGT
MdTNL1OE2	Forward	GGGGTACCATGGATGCTACCATCACAGC
MdTNL1OE2	Reserve	GCGTCGACAGTCAGAAGATCAAACCTTA

MdTNL-1RNAi	Forward	GGGGACAAGTTTGTACAAAAAA-GCAGGCTTCATAAGTGCACCAATCTCAAT
MdTNL-4RNAi	Reserve	GGGGACCACTTTGTACAAGAAAGCTGGGTT-GGTTGGACACCCCTGGACAA
MdTNL1RNAi	Forward	ATAAGTGCACCAATCTCAAT
MdTNL1RNAi	Reserve	GGTTGGACACCCCTGGACAA
qMdTNL1	Forward	GAGTCGTTTGGTCGAGGGAG
qMdTNL1	Reserve	CAAACCTCCACAGTAATCAA
qMdactin	Forward	ATGCCAGGGAACATGGTAGA
qMdactin	Reserve	TGAGCGAGAAATTGTCAGGG
SNP$_{7309212}$-A1	Forward	GAAGGTGAC-CAAGTTCATGCTCGAACTCCAATTTAA-TAAGTGATGCAA
SNP$_{730921}$-A2	Reserve	GAAGGTCGGAGTCAACGGAT-TCGAACTCCAATTTAATAAGTGATGCAT
SNP$_{730921}$-C	Forward	ACTTCTAAAGAAGATATAGACCCGAGATC
1300	Forward	GGGATGACGCACAATCCCAC
PCRSNP$_{7309212}$	Forward	AGATTGATACTCAGAGACTG
PCRSNP$_{7309212}$	Reserve	CAATGAAGGTCCAGGAAGAA
HRMSNP$_{7309212}$	Forward	TCTCTCTCTTGCGAACTCCA
HRMSNP$_{7309212}$	Reserve	CAGTGAGGATGGGTAGTGCT
